# Association between A body shape index and bone mineral density in middle-aged and elderly adults: a retrospective analysis of NHANES 2005–2018

**DOI:** 10.3389/fendo.2025.1506841

**Published:** 2025-04-07

**Authors:** Zhi-Zhuang Wang, Guo-Liang Ma, Bo Xu, Xin Chen, Bo-Wen Yang, Xiao-Kuan Qin, Wei-Li Duan, Min-Shan Feng, He Yin, Kai Sun, Li-Guo Zhu

**Affiliations:** ^1^ Department of Spine, Wangjing Hospital, China Academy of Chinese Medical Sciences, Beijing, China; ^2^ Nanyang Hospital, Wangjing Hospital, Chinese Academy of Traditional Chinese Medicine (Dushan Hospital District), Henan, China; ^3^ Nanyang Key Laboratory of Orthopedic Biomechanics of Traditional Chinese Medicine, Henan, China; ^4^ Beijing Key Laboratory of Bone Setting Technology of Traditional Chinese Medicine, Beijing, China

**Keywords:** bone mineral density, osteoporosis, abdominal obesity, A body shape index, NHANES

## Abstract

**Introduction:**

Despite accumulating evidence on central obesity and osteoporosis, the role of a body shape index (ABSI), a nonlinear index quantifying body shape via body mass index (BMI), waist circumference (WC), and height, remains controversial and underexplored. Although recent meta-analyses suggest central obesity may modulate fracture risk bidirectionally, no research has comprehensively compared ABSI with traditional adiposity metrics, such as BMI, WC, and waist-to-height ratio (WHtR), to predict site-specific changes in bone mineral density (BMD) across anatomical regions.

**Methods:**

This study utilized National Health and Nutrition Examination Survey (NHANES) data from 2005 to 2018, involving 12,421 participants. ABSI was computed using the formula: ABSI = WC/(BMI²/³ × Height¹/²). BMD was assessed at four sites—the total femur (TF), femoral neck (FN), trochanter (TR), and intertrochanter (IN) regions—via dual-energy X-ray absorptiometry (DXA). The association between ABSI and BMD was analyzed via multiple regression models and a generalized additive model (GAM). To compare ABSI’s predictive efficacy with conventional adiposity indices, regression analyses juxtaposed ABSI against BMI, WC, and WHtR in assessing correlations with site-specific BMD.

**Results:**

After full covariate adjustment, a significant negative association was observed between ABSI and BMD in four femoral regions (*P*< 0.01). Smoothed curve fitting revealed a significant nonlinear relationship and threshold effect between ABSI and BMD among middle-aged and older individuals. Additionally, an inverted J-shaped curve was observed between ABSI and BMD in all four femoral regions. Meanwhile, ABSI showed significant negative associations with BMD across all femoral sites (β = -0.27 to -0.31, *p*-trend< 0.000001), whereas BMI, WC, and WHtR exhibited positive correlations (WHtR showing the strongest effect: β = 0.41–0.69). This highlights ABSI’s ability to detect central adiposity-related bone loss obscured by conventional obesity metrics.

**Conclusion:**

ABSI’s robust inverse associations with femoral BMD (β = -0.27 to -0.31), persisting across nonlinear threshold analyses, establish it as a novel biomarker of central adiposity-related skeletal fragility. Unlike conventional indices reflecting mechanical loading benefits (BMI β = 0.008–0.012; WC β = 0.003–0.005; WHtR β = 0.41–0.69), ABSI specifically captures visceral fat-driven metabolic disorder—a critical pathway for osteoporosis risk stratification in normal-weight and obese populations.

## 1 Introduction

Osteoporosis is a disorder of decreased bone mass, microarchitectural deterioration, and fragility fractures (1). As BMD declines, the incidence of fractures increases, particularly among older adults (2). The rising prevalence of osteoporosis and its associated risk of bone fractures have emerged as significant public health issues, driven by the aging global population (3). Preventing osteoporosis presents significant challenges within contemporary healthcare, underscoring the urgent need to investigate its underlying causes to mitigate its global impact (4). Osteoporosis is a complex, chronic condition influenced by age, genetics, and ecological factors. It exhibits substantial variability, and existing standard diagnostic techniques often fall short of accurately identifying all individuals at risk of osteoporotic fractures and providing appropriate treatment (5, 6). Traditional adiposity metrics, such as BMI, WC, and WHtR, have long been used to assess osteoporosis risk but are increasingly recognized for their limitations in specificity and sensitivity (7). BMI, while positively correlated with BMD through mechanical loading, cannot differentiate between fat and muscle mass, which have opposing effects on bone health (8). WC, a measure of central obesity, lacks height normalization, leading to underestimation of visceral adiposity in shorter individuals (9). WHtR, despite its linear height adjustment, fails to account for nonlinear fat distribution patterns, particularly in extreme height groups (10). These limitations are compounded by the reliance on mechanical loading effects, which may mask the metabolic impacts of visceral fat on bone microstructure (11). ABSI’s mathematical formulation addresses the limitations of WC and BMI. First, the nonlinear height scaling reduces bias in shorter/taller individuals, unlike linear adjustments in WHtR (12). Second, the ABSI decouples central adiposity from total body mass, enabling it to isolate visceral fat’s metabolic effects (13). Clinically, ABSI correlates more strongly with VAT and inflammatory markers than WC or WHtR (14), explaining its unique relationship with BMD—a pattern of relationship superior to traditional metrics that conflate mechanical and metabolic effects. Novel metrics like ABSI, which nonlinearly adjusts waist circumference for body size, may better capture the metabolic toxicity of visceral adiposity and improve risk prediction (15). Consequently, there is growing interest in identifying novel risk factors or biomarkers to more accurately assess osteoporosis risk and explore new prevention strategies.

Obesity is a condition characterized by excess body fat that adversely affects health and is strongly associated with various comorbidities, including diabetes and cardiovascular disease (16). Additionally, a significant relationship has been observed between obesity and osteoporosis (17, 18). BMI has traditionally been the primary metric for assessing obesity (19). A multitude of studies have demonstrated a significant positive relationship between BMI and BMD (20). However, some researchers argue that BMI, as a measure of overall obesity, does not effectively differentiate between generalized and central obesity (21). Evidence suggests that central obesity may negatively impact bone development in middle-aged and elderly adults (22). Nonetheless, there is a lack of effective indicators to specifically assess the relationship between central obesity and BMD in this population.

ABSI was introduced by Krakauer et al. (12) in 2012 as a novel anthropometric measure derived from height, weight, and BMI. Unlike BMI, which cannot differentiate between fat and muscle mass (23), ABSI serves as an indicator of central obesity and more accurately assesses adiposity (24, 25). ABSI has been significantly associated with conditions such as diabetes (26, 27) and hypertension (28, 29). Additionally, it provides a more precise assessment of the relationship between central obesity and BMD compared to BMI (30). Sun et al. (22) demonstrated a nonlinear relationship between visceral obesity and BMD via a GAM with a smoothing curve fitting (SCF); however, research specifically exploring the association between ABSI and BMD among middle-aged and older adults remains limited (31). Consequently, there is an urgent need for new indicators to elucidate this relationship. This research seeks to explore the relationship between ABSI and BMD utilizing data derived from the NHANES conducted in the United States from 2005 to 2018.

## 2 Methods

### 2.1 Data origins and study sample

**Figure 1:**
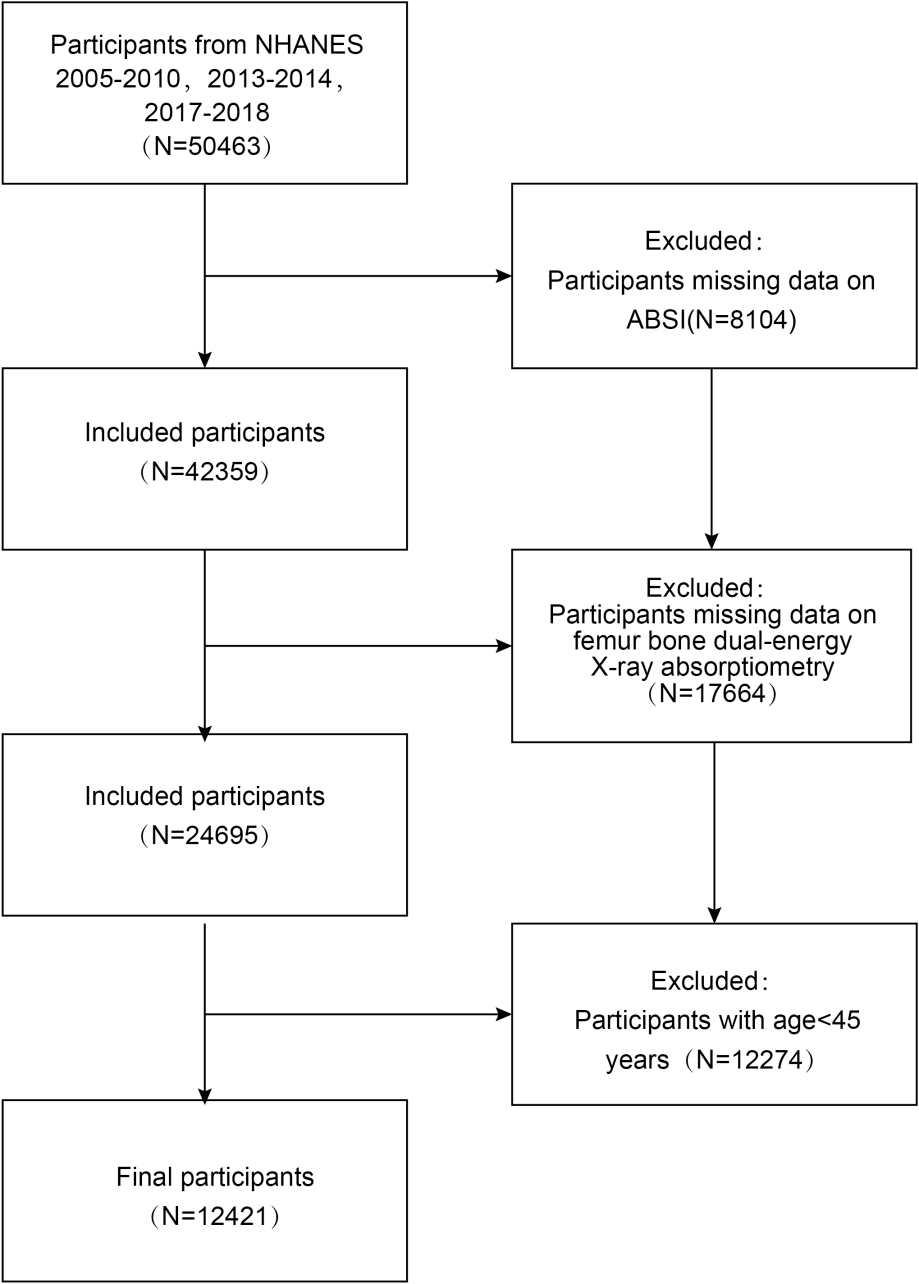
Flow chart of participants selection from the NHANES 2005–2018.

This investigation leverages data derived from the NHANES spanning from 2005 to 2018, employing a cross-sectional design (32). NHANES collects data obtained from a representative cohort of non-institutionalized individuals, selected through a multi-stage, probability-based sampling strategy. This design ensures that the sample accurately reflects the broader U.S. population. The survey is conducted biennially (33). The NHANES research proposal has been authorized by the Ethics Review Committee of the National Center for Health Statistics (NCHS) (34). Written informed consent was secured from all participants prior to their involvement in the study. Data analysis was undertaken from April 1 to April 30, 2024. Details on the NCHS IRB/ERB Protocol Number are available in the **Supplementary Material**. Among the initial cohort of 50,463 participants, exclusions were made for (1) missing ABSI data (n = 8,104), (2) missing femoral BMD data (n = 17,664), and (3) individuals aged less than 45 years (n = 12,274). Consequently, a total of 12,421 participants were incorporated into the final analysis (**Figure 1**).

### 2.2 Predictor variable

ABSI considered the predictor variable, was recently recommended as a measure of body shape based on WC, weight, and height (12). Data for WC (cm), weight (kg), and height (cm) were obtained from the 2005–2018 NHANES dataset. ABSI is calculated using the following formula: WC divided by the product of BMI raised to the two-thirds power and the square root of height (14).


$$ABSI=\frac{WC}{BMI^{2/3}height^{1/2}}$$

### 2.3 Outcome variables

The ending variables measured were BMD at four anatomical sites: the TF, FN, TR, and IN regions. Detailed information regarding the DXA examination techniques can be located within the Body Composition Procedures Manual available on the NHANES website (35).

### 2.4 Covariables

The selection of covariates was guided by theoretical considerations (For example, large missing values for covariates lead to selection bias, as detailed in the **Supplementary Material**) and criteria from existing studies (36, 37). The covariates consisted of sex, age, race, the ratio of family income poverty (PIR), alkaline phosphatase (ALP, IU/L), phosphorus (mg/dL), total calcium (TC, mg/dL), cotinine (ng/mL), energy intake (kcal/day), diabetes, hypertension, and smoking status. Age, PIR, ALP, phosphorus, TC, cotinine, and energy intake were treated as continuous variables, while race, sex, diabetes, hypertension, and smoking status were included as categorical variables. Race was classified into five groups: non-Hispanic White, non-Hispanic Black, Mexican American, other Hispanic, and other. Energy intake (kcal/day) was determined as the mean value from two 24-hour dietary recall assessments. Based on the questionnaire collection, responses for diabetes status were categorized as yes, no, and borderline, and for hypertension status as yes and no. For smoking status, the question was asked, “Do you smoke at least 100 cigarettes in your lifetime” and “Yes or no”.

### 2.5 Statistical analysis

By NCHS analytical guidelines, sample weights were applied to all estimates to accurately characterize the non-institutionalized civilian demographic of the United States (38). To address missing covariate data, multiple imputation techniques were utilized (39). The NHANES database utilizes a sophisticated multistage probability-based sampling methodology (40).

Participant demographics were assessed about ABSI quartiles using Chi-square tests for categorical variables and T-tests for continuous variables. Weighted multivariate logistic regression models were employed to investigate the relationship between ABSI and TF, FN, TR, and IN BMD. By the STROBE guidelines (41), The research employed a set of three models for its analytical framework. Model 1 utilized univariate logistic regression analysis. Model 2 included adjustments for sex, race, and age. Model 3 adjusts age, sex, race, PIR, ALP, phosphorus, total calcium, cotinine, energy, diabetes, hypertension, and smoking status. To explore potential interactions and account for confounding by categorical variables, a subgroup analysis was performed using weighted multivariable logistic regression. We examined the association between ABSI and BMD across various subgroups, stratified by sex, race, age, TC intake, diabetes, hypertension, and smoking status. This detailed subgroup analysis enabled a more nuanced understanding of the relationship between ABSI and BMD within these specific demographic and physiological contexts. The results within different strata are considered valid when the interaction *P*-value is not statistically significant. Conversely, a significant interaction *P*-value indicates the presence of a distinct subgroup within the population, suggesting that the effect of the variables may differ across strata. Previous evidence indicates a nonlinear association between the ABSI and BMD in adolescents (14). Given the elevated prevalence of osteoporosis in middle-aged and older adults, we applied GAM incorporating SCF to NHANES 2005–2018 data to systematically evaluate potential nonlinear ABSI-BMD relationships in this population. This approach allowed us to capture and analyze the complex, non-linear associations between these variables. Significant inflection points in the relationship between ABSI and BMD were identified using a recursive algorithm, following the detection of non-linearity. We then compared a two-part logistic regression model with a traditional logistic regression model incorporating threshold effect analysis to assess the impact of these inflection points. Statistical analyses for this study were performed using R (http://www.r-project.org) and EmpowerStats (http://www.empowerstats.com). All analyses were conducted with a significance threshold set at *P*<0.01.

## 3 Results

### 3.1 Baseline characteristics

In the wake of applying the inclusion and exclusion criteria, the study enrolled 12,421 participants with a mean age of 60.34 ± 10.41 years. The cohort was comprised of 48.82% males and 51.18% females. The average ABSI for the participants was 0.83 ± 0.05. The mean BMD values were 0.93 ± 0.16 g/cm² for the TF, 0.77 ± 0.14 g/cm² for the FN, 0.71 ± 0.13 g/cm² for the TR, and 1.11 ± 0.19 g/cm² for the IN. Compared with the first quartile of the ABSI, the highest quartiles had lower TF, FN, TR, and IN BMD, PIR, phosphorus, and daily energy intake, and higher age, ALP levels, and cotinine (all *P* < 0.05). The prevalence of diabetes, smoking, and hypertension demonstrated a significant upward trend across ABSI quartiles. Specifically, participants in the highest ABSI quartile exhibited a markedly higher prevalence of diagnosed diabetes (20.78%) compared to those in the lowest quartile (7.53%). Similarly, the proportion of individuals with a history of smoking was substantially greater in the highest quartile (59.08%) relative to the lowest quartile (39.06%). Additionally, the prevalence of hypertension increased significantly from 38.97% in the first quartile to 54.56% in the highest quartile. All these trends were statistically significant (*P*< 0.0001). In contrast, total calcium levels did not differ significantly across ABSI quartiles (*P* = 0.1057) (**Table 1**).

**Table 1:** Weighted characteristics of the study population based on ABSI quartiles.

Characteristics	ABSI Quartiles					*P*-value
	Overall	Q1	Q2	Q3	Q4	
Participants	12421	3105	3105	3105	3106	
Age(years)	60.34 ± 10.41	56.42 ± 9.15	58.56 ± 9.65	61.12 ± 9.97	66.42 ± 10.30	<0.0001
Sex (%)						<0.0001
Male	48.82	31.27	51.49	58.54	56.99	
Female	51.18	68.73	48.51	41.46	43.01	
Race/ethnicity (%)						<0.0001
Mexican American	5.40	4.87	6.03	5.72	5.00	
Other Hispanic	3.98	4.12	4.22	4.38	3.11	
Non-Hispanic White	74.90	71.16	72.59	76.28	80.69	
Non-Hispanic Black	9.40	14.01	10.22	7.35	4.96	
Other Race	6.32	5.84	6.95	6.26	6.23	
PIR	3.22 ± 1.55	3.43 ± 1.53	3.35 ± 1.55	3.17 ± 1.56	2.89 ± 1.50	<0.0001
ALP (IU/L)	71.26 ± 23.49	68.31 ± 21.26	70.97 ± 23.61	71.70 ± 22.79	74.80 ± 26.11	<0.0001
Phosphorus (mg/dl)	3.74 ± 0.55	3.78 ± 0.53	3.70 ± 0.56	3.73 ± 0.57	3.74 ± 0.55	<0.0001
Total calcium(mg/dl)	9.43 ± 0.36	9.43 ± 0.36	9.42 ± 0.36	9.43 ± 0.35	9.44 ± 0.37	0.1057
Cotinine (ng/ml)	52.89 ± 125.79	38.92 ± 106.81	50.98 ± 124.87	65.25 ± 138.83	58.98 ± 131.78	<0.0001
Energy (kcal/day)	1988.50 ± 721.41	1935.73 ± 703.06	2042.69 ± 738.08	2044.21 ± 759.32	1930.66 ± 670.73	<0.0001
Total femur BMD(g/cm^2^)	0.93 ± 0.16	0.94 ± 0.16	0.95 ± 0.16	0.94 ± 0.16	0.90 ± 0.15	<0.0001
Femoral neck BMD(g/cm^2^)	0.77 ± 0.14	0.79 ± 0.14	0.79 ± 0.14	0.77 ± 0.14	0.73 ± 0.13	<0.0001
Trochanter BMD(g/cm^2^)	0.71 ± 0.13	0.71 ± 0.13	0.73 ± 0.13	0.71 ± 0.14	0.68 ± 0.13	<0.0001
Intertrochanter BMD(g/cm^2^)	1.11 ± 0.19	1.11 ± 0.19	1.13 ± 0.19	1.12 ± 0.19	1.07 ± 0.19	<0.0001
Diabetes, (%)						<0.0001
Yes	13.31	7.53	11.42	15.04	20.78	
No	83.73	89.92	86.01	81.70	75.62	
Borderline	2.96	2.55	2.57	3.26	3.60	
Smoke						<0.0001
Yes	48.95	39.06	46.53	53.52	59.08	
No	51.05	60.94	53.47	46.48	40.92	
High blood pressure						<0.0001
Yes	45.30	38.97	41.54	48.01	54.56	
No	54.70	61.03	58.46	51.99	45.44	

ABSI, A body shape index; BMD, Bone mineral density; PIR, ratio of family income poverty; ALP, Alkaline phosphatase

Mean ± SD for continuous variables: the *P-*value was calculated by the weighted linear regression model. (percentage) for categorical variables: the *P-*value was calculated by the weighted chi-square test.

### 3.2 Association of ABSI, BMI, WC, and WHtR with BMD

We assessed the association between ABSI and BMD at four femoral measurement sites by treating ABSI both as a continuous variable and as quartiles, employing weighted multiple logistic regression models for analysis. We further conducted multiple regression analyses to separately evaluate the associations of ABSI, BMI, WC, and WHtR with BMD. All models demonstrated a negative association between ABSI and BMD. After adjusting for all covariates, a one-unit increase in ABSI was associated with reductions in BMD at the TF, FN, TR, and IN sites. Specifically, the reductions were 0.29 g/cm² for the TF, 0.32 g/cm² for the FN, 0.28 g/cm² for the TR, and 0.29 g/cm² for the IN region. Additionally, comparing the highest and lowest ABSI quartiles, BMD decreased by 0.03 g/cm² at the TF, FN, TR, and IN sites (**Table 2**). The associations between ABSI, BMI, WC, and WHtR with BMD at different skeletal sites were evaluated using multiple regression analysis, and the results are presented in **Table 3**. After adjusting for all covariates, ABSI was found to be significantly and negatively associated with BMD at all measured sites. Specifically, each unit increase in ABSI was associated with a decrease in BMD of 0.2904 g/cm² (95% CI: -0.3485, -0.2324) for the TF, 0.3084 g/cm² (95% CI: -0.3619, -0.2549) for the FN, and 0.2726 g/cm² (95% CI: -0.3232, -0.2220) for the TR. Additionally, the BMD of the IN region decreased by 0.3007 g/cm² (95% CI: -0.3706, -0.2307), with all trends reaching statistical significance (*P*< 0.05). In contrast, BMI, WC, and WHtR were positively associated with BMD at all sites, with WHtR demonstrating the strongest effect size. For example, WHtR was positively associated with TF BMD (β = 0.5746, 95% CI: 0.5462, 0.6029), FN BMD (β = 0.4111, 95% CI: 0.3843, 0.4378), TR BMD (β = 0.4162, 95% CI: 0.3910, 0.4413), and IN BMD (β = 0.6895, 95% CI: 0.6555, 0.7236) (*P*< 0.05) (**Table 3**). These findings suggest that while general measures of adiposity, such as BMI, WC, and WHtR, are positively correlated with BMD, a higher ABSI (reflecting a more centralized fat distribution) may be associated with lower BMD, indicating that ABSI may have an adverse effect on bone health.

**Table 2:** Association of BMD with ABSI in different models among all participants.

Predictor variable	Model 1 [β (95%CI)]	Model 2 [β (95%CI)]	Model 3 [β (95%CI)]
Total femur BMD (continuous)	-0.37 (-0.45, -0.30)	-0.34 (-0.40, -0.27)	-0.29 (-0.36, -0.22)
Total femur BMD (quartile)			
Q1	Reference	Reference	Reference
Q2	0.02 (0.01, 0.02)	-0.00 (-0.01, 0.00)	0.00 (-0.00, 0.01)
Q3	0.00 (-0.01, 0.01)	-0.01 (-0.02, -0.01)	-0.01 (-0.02, -0.00)
Q4	-0.04 (-0.05, -0.03)	-0.04 (-0.04, -0.03)	-0.03 (-0.04, -0.02)
*p* for trend	<0.0001	<0.0001	<0.0001
Femoral neck BMD (continuous)	-0.56 (-0.62, -0.50)	-0.35 (-0.41, -0.28)	-0.32 (-0.39, -0.26)
Femoral neck BMD (quartile)			
Q1	Reference	Reference	Reference
Q2	0.00 (-0.01, 0.01)	-0.01 (-0.01, 0.00)	-0.00 (-0.01, 0.00)
Q3	-0.02 (-0.03, -0.01)	-0.02 (-0.02, -0.01)	-0.02 (-0.02, -0.01)
Q4	-0.06 (-0.07, -0.05)	-0.04 (-0.04, -0.03)	-0.03 (-0.04, -0.02)
*p* for trend	<0.0001	<0.0001	<0.0001
Trochanter BMD (continuous)	-0.30 (-0.36, -0.24)	-0.34 (-0.40, -0.28)	-0.28 (-0.34, -0.22)
Trochanter BMD (quartile)			
Q1	Reference	Reference	Reference
Q2	0.01 (0.01, 0.02)	-0.00 (-0.01, 0.00)	-0.00 (-0.01, 0.01)
Q3	0.00 (-0.00, 0.01)	-0.02 (-0.02, -0.01)	-0.01 (-0.02, -0.01)
Q4	-0.03 (-0.04, -0.03)	-0.04 (-0.04, -0.03)	-0.03 (-0.04, -0.02)
*p* for trend	<0.0001	<0.0001	<0.0001
Intertrochanter BMD (continuous)	-0.38 (-0.47, -0.30)	-0.33 (-0.42, -0.25)	-0.29 (-0.37, -0.20)
Intertrochanter BMD (quartile)			
Q1	Reference	Reference	Reference
Q2	0.02 (0.01, 0.03)	-0.00 (-0.01, 0.01)	0.00 (-0.00, 0.01)
Q3	0.01 (-0.00, 0.01)	-0.01 (-0.02, -0.01)	-0.01 (-0.02, -0.00)
Q4	-0.04 (-0.05, -0.03)	-0.04 (-0.04, -0.03)	-0.03 (-0.04, -0.02)
*p* for trend	<0.0001	<0.0001	<0.0001

ABSI, A body shape index; BMD, Bone mineral density; CI, confidence intervals; SD, standard deviation

Model 1: no covariates were adjusted. **Model 2:** age, sex, and race were adjusted. **Model 3:** age, sex, race, PIR, ALP, phosphorus, total calcium, cotinine, energy, diabetes, hypertension, and smoking status were adjusted.

**Table 3:** Correlation of ABSI, BMI, WC and WHtR with BMD.

	ABSI [β (95%CI)]	BMI [β (95%CI)]	WC [β (95%CI)]	WHtR [β (95%CI)]
Total femur BMD	-0.2904(-0.3485, -0.2324)	0.0104(0.0100, 0.0108)	0.0039(0.0037, 0.0040)	0.5746(0.5462, 0.6029)
Femoral neck BMD	-0.3084(-0.3619, -0.2549)	0.0080(0.0076, 0.0083)	0.0029(0.0027, 0.0030)	0.4111(0.3843, 0.4378)
Trochanter BMD	-0.2726(-0.3232, -0.2220)	0.0078(0.0075, 0.0082)	0.0029(0.0027, 0.0030)	0.4162(0.3910, 0.4413)
Intertrochanter BMD	-0.3007(-0.3706, -0.2307)	0.0122(0.0117, 0.0126)	0.0045(0.0043, 0.0047)	0.6895(0.6555, 0.7236)
*p* for trend	<0.000001	<0.000001	<0.000001	<0.000001

ABSI, A body shape index; BMI, Body mass index; WC, Waist circumference; WHtR, Waist-to-height ratio; BMD, Bone mineral density; CI, confidence intervals; SD, standard deviation

Age, sex, race, PIR, ALP, phosphorus, total calcium, cotinine, energy, diabetes, hypertension, and smoking status were adjusted.

### 3.3 Subgroup analysis

The subgroup analyses were conducted to examine the robustness of the association between the ABSI and BMD at TF, FN, TR, and IN regions across different demographic and clinical subgroups. Missing data rates for covariates were as follows: alcohol consumption (76.64%), physical activity (15.31%), and no missing data for smoking status, diabetes, or hypertension. It has been shown that the exclusion of covariates with missing values of more than 10% reduces the potential bias of the data (42), while Groenwold et al. suggested that a high rate of missingness may distort the associations in the study and cause selection bias (43). This study strictly excluded covariates with missing values greater than 10% in order to avoid this situation and reduce potentially misleading results. Therefore, as suggested by observational data, we prioritized analytic rigor by excluding these variables (e.g., alcohol consumption and physical activity) from subgroup analyses. Significant interactions were observed in the sex-stratified analysis (*P* for interaction< 0.0001) across all four BMD sites, indicating that sex significantly modifies the relationship between ABSI and femoral BMD. Specifically, the negative association was stronger in males, with β values of -0.54 (95% CI: -0.64, -0.45) for TF, -0.48 (95% CI: -0.57, -0.39) for FN, -0.49 (95% CI: -0.58, -0.41) for TR, and -0.59 (95% CI: -0.71, -0.47) for IN. In contrast, the association was attenuated in females, with β values of -0.18 (95% CI: -0.25, -0.11) for TF, -0.23 (95% CI: -0.30, -0.17) for FN, -0.17 (95% CI: -0.24, -0.11) for TR, and -0.17 (95% CI: -0.25, -0.08) for IN. Regarding race/ethnicity, the interaction analysis revealed statistically significant differences in the associations of ABSI with BMD at the TF (*P* = 0.0640), TR (*P* = 0.0563), and IN (*P* = 0.0506) regions, whereas the relationship at the FN site was not significantly modified by race (*P* = 0.2909). Among racial groups, Mexican Americans exhibited the strongest negative association, with β values of -0.60 (95% CI: -0.85, -0.34) for TF, -0.51 (95% CI: -0.74, -0.27) for FN, -0.54 (95% CI: -0.77, -0.32) for TR, and -0.68 (95% CI: -0.99, -0.38) for IN. Non-Hispanic Whites showed a more moderate association, with β values ranging from -0.24 to -0.28 across different sites, while non-Hispanic Blacks and individuals of other races exhibited comparable trends with slight variations. Age-stratified analyses did not show significant interactions (*P* for interaction > 0.05), suggesting that the association between ABSI and BMD remained relatively consistent across different age groups. However, a trend was observed where the negative association appeared most pronounced in individuals aged 62–70 years, with β values of -0.40 (95% CI: -0.52, -0.28) for TF, -0.43 (95% CI: -0.54, -0.32) for FN, -0.36 (95% CI: -0.47, -0.26) for TR, and -0.41 (95% CI: -0.55, -0.26) for IN. Similarly, no significant interactions were observed for total calcium intake, suggesting that dietary calcium levels did not substantially modify the association between ABSI and BMD. Subgroup analysis based on diabetes status revealed a statistically significant interaction (*P* for interaction< 0.01) across all four femoral sites, indicating that the presence of diabetes significantly modifies the relationship between ABSI and BMD. Individuals with diabetes exhibited a stronger negative association (β = -0.57 to -0.63 across different sites) compared to those without diabetes (β = -0.23 to -0.27). Those classified as borderline diabetes also demonstrated a comparable negative trend. Regarding smoking status, the interaction analysis did not reveal significant differences (*P* for interaction > 0.05), indicating that the association between ABSI and BMD was similar in both smokers and non-smokers. Likewise, hypertension status did not significantly modify the association at any femoral site (*P* > 0.05) (**Table 4**).

**Table 4:** Subgroup analysis of the associations between ABSI and BMD.

Subgroup	Total femur BMD [β(95%CI)]	*P* for interaction	Femoral neck BMD[β(95%CI)]	*P* for interaction	Trochanter BMD[β(95%CI)]	*P* for interaction	Intertrochanter BMD[β(95%CI)]	*P* for interaction
Sex		<0.0001		<0.0001		<0.0001		<0.0001
Male	-0.54 (-0.64, -0.45)		-0.48 (-0.57, -0.39)		-0.49 (-0.58, -0.41)		-0.59 (-0.71, -0.47)	
Female	-0.18 (-0.25, -0.11)		-0.23 (-0.30, -0.17)		-0.17 (-0.24, -0.11)		-0.17 (-0.25, -0.08)	
Race/ethnicity		0.0640		0.2909		0.0563		0.0506
Mexican American	-0.60 (-0.85, -0.34)		-0.51 (-0.74, -0.27)		-0.54 (-0.77, -0.32)		-0.68 (-0.99, -0.38)	
Other Hispanic	-0.42 (-0.71, -0.12)		-0.36 (-0.63, -0.09)		-0.44 (-0.70, -0.19)		-0.40 (-0.75, -0.04)	
Non-Hispanic White	-0.25 (-0.32, -0.19)		-0.28 (-0.34, -0.22)		-0.24 (-0.30, -0.18)		-0.25 (-0.33, -0.17)	
Non-Hispanic Black	-0.34 (-0.52, -0.16)		-0.40 (-0.56, -0.23)		-0.28 (-0.44, -0.12)		-0.37 (-0.59, -0.15)	
Other Race	-0.40 (-0.63, -0.16)		-0.34 (-0.56, -0.12)		-0.34 (-0.55, -0.14)		-0.47 (-0.75, -0.18)	
Age		0.6013		0.4504		0.2607		0.7503
45 - 52 years old	-0.32 (-0.43, -0.21)		-0.33 (-0.43, -0.22)		-0.35 (-0.44, -0.25)		-0.32 (-0.45, -0.18)	
53 - 61 years old	-0.33 (-0.43, -0.22)		-0.32 (-0.42, -0.23)		-0.27 (-0.37, -0.18)		-0.37 (-0.49, -0.24)	
62 - 70 years old	-0.40 (-0.52, -0.28)		-0.43 (-0.54, -0.32)		-0.36 (-0.47, -0.26)		-0.41 (-0.55, -0.26)	
71 - 85 years old	-0.29 (-0.41, -0.17)		-0.33 (-0.43, -0.22)		-0.24 (-0.34, -0.13)		-0.31 (-0.45, -0.17)	
Total calcium		0.7297		0.5392		0.4741		0.7666
Q1	-0.33 (-0.46, -0.21)		-0.33 (-0.45, -0.22)		-0.32 (-0.43, -0.21)		-0.35 (-0.51, -0.20)	
Q2	-0.28 (-0.41, -0.16)		-0.32 (-0.44, -0.21)		-0.31 (-0.43, -0.20)		-0.27 (-0.43, -0.12)	
Q3	-0.32 (-0.43, -0.21)		-0.35 (-0.45, -0.25)		-0.27 (-0.37, -0.18)		-0.33 (-0.46, -0.20)	
Q4	-0.25 (-0.35, -0.16)		-0.26 (-0.35, -0.18)		-0.23 (-0.31, -0.15)		-0.27 (-0.38, -0.16)	
Diabetes, (%)		0.0002		0.0088		0.0010		0.0004
Yes	-0.57 (-0.72, -0.41)		-0.49 (-0.63, -0.35)		-0.48 (-0.61, -0.35)		-0.63 (-0.82, -0.45)	
No	-0.24 (-0.30, -0.18)		-0.27 (-0.33, -0.22)		-0.23 (-0.29, -0.18)		-0.24 (-0.32, -0.17)	
Borderline	-0.48 (-0.80, -0.16)		-0.48 (-0.78, -0.19)		-0.48 (-0.76, -0.21)		-0.46 (-0.84, -0.08)	
Smoke		0.3575		0.7359		0.9231		0.2304
Yes	-0.32 (-0.40, -0.24)		-0.32 (-0.40, -0.24)		-0.28 (-0.35, -0.20)		-0.35 (-0.45, -0.25)	
No	-0.27 (-0.34, -0.19)		-0.30 (-0.37, -0.23)		-0.27 (-0.34, -0.20)		-0.26 (-0.36, -0.17)	
High blood pressure		0.1679		0.0904		0.7402		0.1363
Yes	-0.33 (-0.41, -0.25)		-0.35 (-0.43, -0.28)		-0.28 (-0.35, -0.21)		-0.35 (-0.45, -0.25)	
No	-0.26 (-0.33, -0.18)		-0.27 (-0.34, -0.20)		-0.27 (-0.33, -0.20)		-0.26 (-0.35, -0.17)	

ABSI, A body shape index; BMD, Bone mineral density; CI, confidence intervals; SD, standard deviation

### 3.4 Non-linear relationships

**Figure 2:**
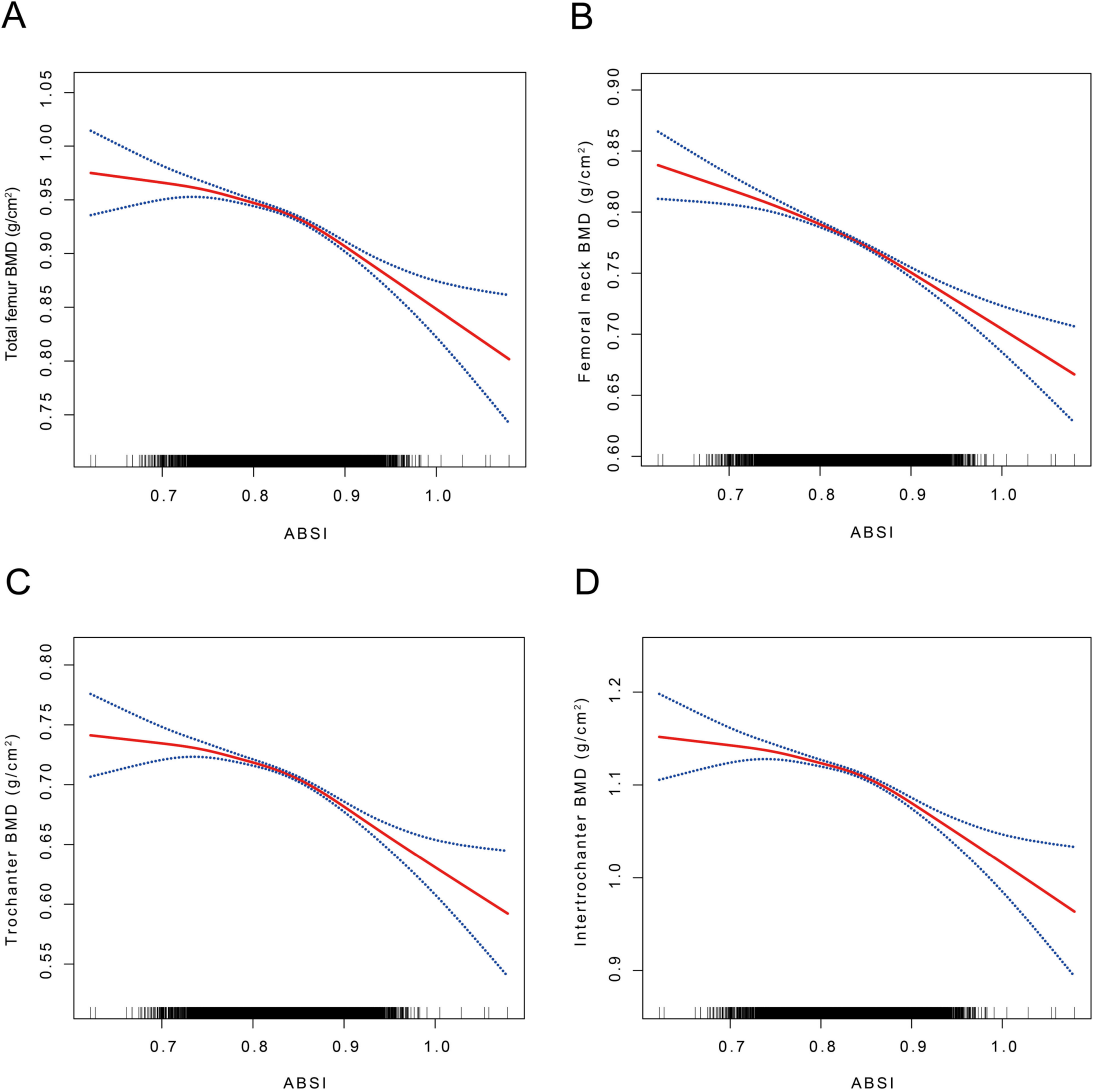
The association between ABSI and TF, FN, TR, and IN BMD. **(A)** The association between ABSI and TF BMD. **(B)** The association between ABSI and FN BMD. **(C)** The association between ABSI and TR BMD. **(D)** The association between ABSI and IN BMD. ABSI, A body shape index; BMD, Bone mineral density; TF BMD, Total femur bone mineral density; FN BMD, Femoral neck bone mineral density; TR BMD, Trochanter bone mineral density; IN BMD, Intertrochanteric bone mineral density A solid red line represents the smooth curve fit between variables. Blue bands represent the 95% confidence interval from the fit. Age, sex, race, PIR, ALP, phosphorus, total calcium, cotinine, energy, diabetes, hypertension, and smoking status were adjusted. SCF using GAM to evaluate the nonlinear relationship between ABSI and TF, FN, TR, and IN BMD.

In this investigation, we employed GAM and SCF techniques to elucidate potential non-linear relationships between the ABSI and BMD. These methods were used to validate and further substantiate our findings. Within the comprehensively adjusted model, an inverted J-shaped relationship was observed between ABSI and BMD at the TF, FN, TR, and IN regions (**Figure 2**). Threshold effect analyses identified inflection points at 0.83, 0.83, 0.83, and 0.84, respectively. Before these inflection points, the correlations between ABSI and BMD in these regions were weakly negative, with odds ratios (ORs) of -0.19 (95% CI: -0.28, -0.09), -0.11 (95% CI: -0.20, -0.02), -0.11 (95% CI: -0.22, -0.00), and -0.09 (95% CI: -0.19, 0.01). Beyond the inflection points, the relationship became statistically significant, showing strong negative correlations, with ORs of -0.41 (95% CI: -0.57, -0.24), -0.25 (95% CI: -0.41, -0.10), -0.33 (95% CI: -0.47, -0.18), and -0.47 (95% CI: -0.68, -0.26), respectively (**Table 5**). The threshold effects were evaluated based on the ABSI for various BMD measurements across all participants. For TF BMD, the threshold ABSI was set at 0.83. When ABSI was below this threshold, the effect value was -0.19; however, when ABSI exceeded 0.83, the effect value was -0.41. For the ABSI-FN BMD, the threshold was determined to be 0.83. Below this threshold, the effect value was -0.11, whereas it changed to -0.25 when ABSI exceeded 0.83. Similarly, for ABSI-TR BMD, the threshold effect value was also 0.83. In this case, the effect value was -0.11 for ABSI values below 0.83 and shifted to -0.33 when ABSI was above 0.83. Finally, for ABSI-IN BMD, the threshold was set at 0.84. The effect value was -0.09 for ABSI values below 0.84 and was significantly to -0.47 when ABSI surpassed 0.84 (**Figure 2** **,** **Table 5**).

**Table 5:** Threshold effect analysis of ABSI on TF, FN, TR and IN BMD.

Outcome	Total femur BMD	Femoral neck BMD	Trochanter BMD	Intertrochanter BMD
Model I				
Fitting by the standard linear model	-0.29 (-0.35, -0.23)<0.0001	-0.31 (-0.36, -0.25)<0.0001	-0.27 (-0.32, -0.22)<0.0001	-0.30 (-0.37, -0.23)<0.0001
Model II				
ABSI inflection point(K)	0.83	0.83	0.83	0.84
< K, effect1	-0.19 (-0.28, -0.09)<0.0001	-0.11 (-0.20, -0.02) 0.0120	-0.11 (-0.22, -0.00) 0.0476	-0.09 (-0.19, 0.01) 0.0725
> K, effect2	-0.41 (-0.57, -0.24)<0.0001	-0.25 (-0.41, -0.10) 0.0016	-0.33 (-0.47, -0.18)<0.0001	-0.47 (-0.68, -0.26)<0.0001
Log-likelihood ratio	<0.001	0.002	<0.001	<0.001

ABSI, A body shape index; BMD, Bone mineral density; TF BMD, Total femur bone mineral density; FN BMD, Femoral neck bone mineral density; TR BMD, Trochanter bone mineral density; IN BMD, Intertrochanter bone mineral density.

Age, sex, race, PIR, ALP, phosphorus, total calcium, cotinine, energy, diabetes, hypertension, and smoking status were adjusted.

**Figure 3:**
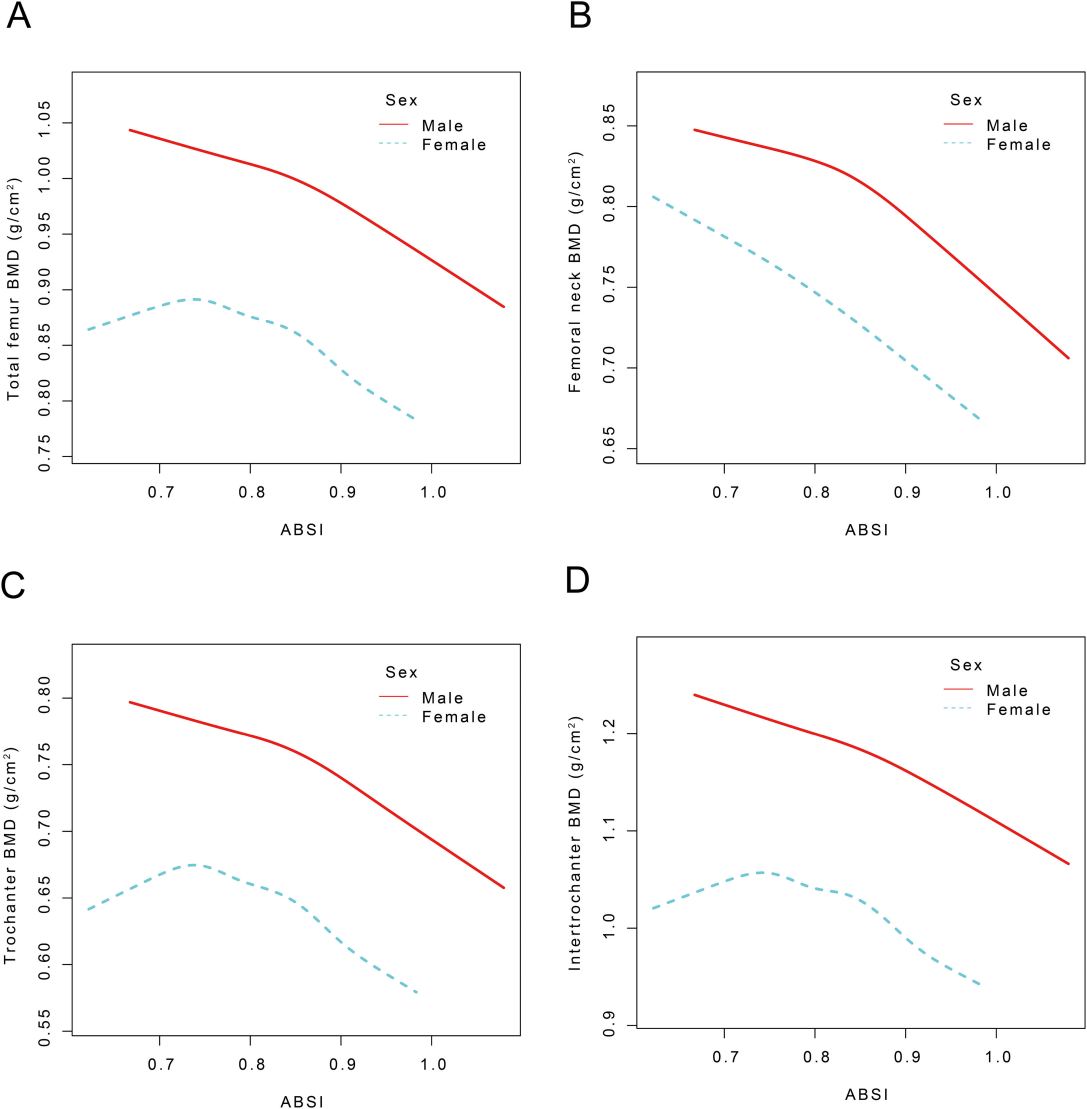
The nonlinear association between ABSI and TF, FN, TR, and IN BMD stratified by sex. ABSI, A body shape index; BMD, Bone mineral density; TF BMD, Total femur bone mineral density, FN BMD, femoral neck bone mineral density; TR BMD, trochanter bone mineral density; IN BMD, intertrochanteric bone mineral density Age, race, PIR, ALP, phosphorus, total calcium, cotinine, energy, diabetes, hypertension, and smoking status were adjusted.

Participants were categorized by sex, and smoothed curve fitting along with threshold effects were assessed to examine the relationships between the ABSI and BMD at various sites including the TF, FN, TR, and IN regions (**Figure 3**). For male participants, no statistically significant correlation was observed between ABSI and TF BMD when ABSI was below 0.81 (β = -0.11, 95% CI: -0.41, 0.18). In contrast, for ABSI values exceeding 0.81, each unit increase in ABSI was linked to a decline in TF BMD of 0.58 g/cm² (95% CI: -0.71, -0.46). In female participants, ABSI showed no significant correlation with TF BMD when ABSI was below 0.85 (β = -0.00, 95% CI: -0.10, 0.10). However, for ABSI values above 0.85, each unit increase in ABSI was linked to a decrease in TF BMD of 0.49 g/cm² (95% CI: -0.69, -0.29). For the ABSI-FN BMD effect, male participants showed no significant correlation when ABSI was below 0.81 (β = -0.04, 95% CI: -0.31,0.23). However, for ABSI values exceeding 0.81, each unit increase in ABSI resulted in a decrease in FN BMD of 0.54 g/cm² (95% CI: -0.66, -0.43). In females, When ABSI is below 0.85, each unit increase in ABSI is indicative of a decrease in FN BMD of 0.14 g/cm² (95% CI: -0.23, -0.05). Conversely, when ABSI exceeds 0.85, each unit increase in ABSI corresponds to a more substantial decrease in FN BMD of 0.33 g/cm² (95% CI: -0.52, -0.15) (*P* < 0.01). Regarding the ABSI-TR BMD effect, no significant correlation was found for male participants when ABSI was below 0.81 (β = -0.08, 95% CI: -0.35, 0.18). Conversely, for ABSI values above 0.81, every unit increase in ABSI was accompanied by a decrease in TR BMD of 0.57 g/cm² (95% CI: -0.68, -0.46). In female participants, ABSI did not significantly correlate with TR BMD when ABSI was below 0.84 (β = -0.04, 95% CI: -0.13, 0.06). When ABSI exceeded 0.84, each unit increase in ABSI was associated with a decrease in TR BMD of 0.36 g/cm² (95% CI: -0.50, -0.21). For the ABSI-IN BMD effect, there was no statistically significant correlation for male participants when ABSI was below 0.81 (β = -0.19, 95% CI: -0.54, 0.17). However, for ABSI values above 0.81, each unit increase in ABSI resulted in a decrease in IN BMD of 0.60 g/cm² (95% CI: -0.75, -0.45). In females, no significant correlation was found when ABSI was below 0.85 (β = 0.05, 95% CI: -0.07, 0.18). For ABSI values exceeding 0.85, every unit increment in ABSI was indicative of a decrease in IN BMD of 0.59 g/cm² (95% CI: -0.83, -0.34) (**Table 6**).

**Table 6:** Threshold effects between ABSI and TF, FN, TR, and IN BMD stratified by sex.

Stratified by sex	Male	Female
Total femur BMD [β(95%CI)]		
ABSI turning point(K)	0.81	0.85
< K, effect1	-0.11 (-0.41, 0.18) 0.4571	-0.00 (-0.10, 0.10) 0.9645
> K, effect2	-0.58 (-0.71, -0.46)<0.0001	-0.49 (-0.69, -0.29)<0.0001
Log-likelihood ratio	0.010	<0.001
Femoral neck BMD [β(95%CI)]		
ABSI turning point(K)	0.81	0.85
< K, effect1	-0.04 (-0.31, 0.23) 0.7736	-0.14 (-0.23, -0.05) 0.0028
> K, effect2	-0.54 (-0.66, -0.43)<0.0001	-0.33 (-0.52, -0.15) 0.0004
Log-likelihood ratio	0.003	0.110
Trochanter BMD [β(95%CI)]		
ABSI turning point(K)	0.81	0.84
< K, effect1	-0.08 (-0.35, 0.18) 0.5361	-0.04 (-0.13, 0.06) 0.4436
> K, effect2	-0.57 (-0.68, -0.46)<0.0001	-0.36 (-0.50, -0.21)<0.0001
Log-likelihood ratio	0.003	0.002
Intertrochanter BMD [β(95%CI)]		
ABSI turning point(K)	0.81	0.85
< K, effect1	-0.19 (-0.54, 0.17) 0.3054	0.05 (-0.07, 0.18) 0.3907
> K, effect2	-0.60 (-0.75, -0.45)<0.0001	-0.59 (-0.83, -0.34)<0.0001
Log-likelihood ratio	0.058	<0.001

ABSI, A body shape index; BMD, Bone mineral density; TF BMD, Total femur bone mineral density; FN BMD, Femoral neck bone mineral density; TR BMD, Trochanter bone mineral density; IN BMD, Intertrochanter bone mineral density.

Age, race, PIR, ALP, phosphorus, total calcium, cotinine, energy, diabetes, hypertension, and smoking status were adjusted.

## 4 Discussion

This study conducted a comprehensive analysis of NHANES data spanning from 2005 to 2018. A comprehensive analysis of cross-sectional data from 12,421 participants revealed a complex correlation between ABSI and BMD (**Table 2**). Our study demonstrated that ABSI was negatively associated with BMD in the TF, FN, TR, and IN regions, indicating that higher ABSI is associated with lower BMD (**Figure 2**).

To our knowledge, this research represents the inaugural investigation into the correlation between the ABSI and BMD in middle-aged and elderly adults. Although BMI is a widely used metric for assessing obesity (20, 44, 45), it has limited capability in distinguishing between visceral or abdominal adiposity and either muscle mass or subcutaneous fat (46). Previous studies have frequently utilized BMI as the primary metric for defining weight, which has led to conclusions suggesting that overweight and obesity may be protective factors for BMD (47, 48). However, this perspective has increasingly come under scrutiny. For example, Rinonapoli et al. (49) highlighted that while an increase in BMI may be associated with higher BMD, it concurrently elevates the risk of fractures. It has also been proposed that Moderate buildup of visceral fat could be advantageous for bone health, whereas excessive visceral fat may lead to negative consequences (22). Lee et al. (50) observed that obesity, while associated with weight gain, may offer minimal benefit to bone health and could increase the risk of osteoporotic diseases. Antonopoulos et al. (51) emphasized that BMI evaluates obesity based solely on height and weight, without accounting for variations in fat and muscle composition. Some researchers have observed that abdominal obesity is associated with reduced vertebral bone density, thereby increasing the risk of vertebral fractures (52). Krishnan et al. (53) also reported a negative correlation between abdominal obesity and BMD. Therefore, there is a need for a comprehensive index that accurately assesses the relationship between abdominal obesity and BMD in middle-aged and older adults. The ABSI is a novel metric introduced in recent years for evaluating abdominal fat (54). Although the ABSI is derived from height, WC, and BMI, its innovative presentation minimizes the confounding effects of these traditional measurements, thus providing a more refined and accurate assessment of central adiposity (55). ABSI has shown robust predictive capability for various health risks, including liver disease (56), diabetes (57), and cardiovascular conditions (13). Deng et al. (25) investigated the association between BMI, ABSI, and BMD in middle-aged and older adults through a cross-sectional study conducted within a Chinese population. However, to date, no studies have investigated the association between ABSI and BMD specifically in middle-aged and older adults within the U.S. population. To tackle this issue, we carried out a cross-sectional study with a substantial study population to investigate the relationship between ABSI and BMD.

Our findings reveal a significant negative correlation between ABSI and BMD across all four femoral regions, with evidence of a threshold effect. Specifically, the identified threshold values were 0.83, 0.83, 0.83, and 0.84, respectively (**Table 5**). Notably, the negative correlations in these regions were modest before reaching the threshold values but became markedly stronger thereafter (**Figure 2**, **Table 5**). The outcomes of the multiple regression analysis showed that the models showed a negative correlation between ABSI and TF, FN, TR, and IN BMD, which aligns with the findings of Zhang et al. (31). However, our study extends this finding and further reveals the complexity of the relationship between ABSI and BMD. Subgroup analyses indicated a statistically significant interaction by sex regarding the relationship between ABSI and BMD at the TF, FN, TR, and IN regions (interaction *P<* 0.0001). By analyzing data from 40,115 participants in the NHANES database spanning 2007 to 2018 and including 1,557 adolescents, Lin et al. (14) used restricted cubic splines (RCS) curves and subgroup analyses to determine that, except for the FN, BMD at the other three sites demonstrated a strong linear association with ABSI in both male and female groups. There are close parallels with our results. The observed discrepancies may be due to variations in covariates and differences in the age distributions of the studied populations.

A negative correlation between ABSI and BMD has been observed, which may be partly explained by the mechanisms underlying the relationship between central obesity (as reflected by, WC) and bone metabolism (58). Central obesity, as indicated by increased WC, is a key component of ABSI and has been associated with adverse metabolic profiles, including insulin resistance and chronic inflammation (59). These metabolic disturbances can negatively impact bone health through several pathways. Firstly, bone marrow stromal stem cells, which are the primary source of both osteoblasts and adipocytes, can be influenced by obesity-related factors (60). Obesity enhances the differentiation of stromal stem cells into adipocytes, leading to an increase in adipocyte numbers within the bone marrow and a simultaneous decrease in osteoblast formation (61, 62). This shift in cell differentiation can reduce bone formation and increase bone resorption, contributing to lower BMD (63). Secondly, chronic inflammation associated with central obesity is a crucial factor in disrupting bone metabolic homeostasis (64). Elevated levels of pro-inflammatory cytokines, such as TNFα and IL-6, which are commonly observed in obesity, can promote osteoclastogenesis through an NFκB-mediated pathway (65). This process enhances the expression of c-fms, RANK, and RANKL, leading to increased bone resorption (66). Additionally, individuals with central obesity, as indicated by higher WC, often exhibit elevated levels of peroxisome proliferator-activated receptor gamma (PPARγ) metabolites (67). These metabolites are associated with greater adipose infiltration into the bone marrow and decreased differentiation of common progenitor cells into osteoblasts (68). This further contributes to reduced bone formation and lower BMD. In summary, the negative correlation between ABSI and BMD can be explained by the adverse effects of central obesity, as reflected by WC, on bone metabolism through mechanisms involving stem cell differentiation, chronic inflammation, and adipose tissue infiltration into the bone marrow.

The present study revealed a significant inverse association between ABSI and femoral BMD across all anatomical regions, contrasting with the positive correlations observed for conventional adiposity indices including BMI, WC, and WHtR (**Table 3**). This divergence suggests that ABSI may quantify distinct pathophysiological pathways, particularly those involving visceral adipose tissue (VAT)-mediated metabolic dysregulation and aberrant bone remodeling (69). The enhanced sensitivity of ABSI in detecting abnormal fat distribution patterns stems from its nonlinear normalization of waist circumference through the equation WC/(BMI^2/3^ × height^1/2^), which mathematically disentangles central adiposity from overall body size (12). Meanwhile, the mathematical construction of the ABSI decouples changes in muscle-fat ratio in weight gain through fractal dimension adjustment, a property that enables it to reflect both central obesity intensity (waist circumference) and body size scaling effects (height-weight relationship), overcoming the insensitivity of BMI to fat distribution as well as the overdependence of the WHtR on the linear height Limitations of standardization (12, 70, 71). Such computational innovation addresses inherent limitations of traditional anthropometric measures that either conflate fat and lean mass (as in BMI) or inadequately adjust for stature variations (as in WHtR). Notably, the paradoxical association pattern - wherein ABSI demonstrates negative BMD relationships while conventional indices show positive associations - may reflect clinically relevant heterogeneity within obesity phenotypes. Specifically, elevated ABSI values could signal a metabolically obese normal-weight (MONW) profile (72), characterized by disproportionate VAT accumulation. Mechanistically, this ectopic fat depot may impair bone microstructure through dual pathways: (i) systemic inflammation mediated by adipocytokines (e.g., IL-6, TNF-α) that disrupt osteoblast-osteoclast coupling (73), and (ii) altered biomechanical loading patterns due to abnormal body fat distribution (74).

This study analyzed data encompassing not only a single variable but also BMD measurements from different regions, including TF, FN, TR, and IN areas. A stratified analysis was performed to investigate the relationship between ABSI and BMD, accounting for multiple factors. Confounding variables were meticulously assessed, and statistical accuracy was enhanced by identifying and addressing possible confounders. Nonlinear associations were further investigated using SF curves and logistic regression.

Our study presents several limitations. Firstly, being a cross-sectional study, it cannot determine causality between ABSI and BMD in middle-aged and elderly adults. Additionally, due to data limitations, we were unable to account for all potential covariates that may affect bone metabolism, and thus, we cannot completely exclude the possibility that confounding variables may have influenced our results. Nonetheless, the substantial sample size and the use of a nationally representative dataset lend robustness to our findings.

## 5 Conclusion

This study elucidates a distinct correlation pattern between obesity and BMD, indicating that traditional adiposity indices (BMI, WC, WHtR) predominantly reflect the influence of mechanical loading on bone mass accrual, whereas ABSI appears to encapsulate the metabolic ramifications of visceral adiposity. The dose-dependent inverse association between ABSI and femoral BMD (β = -0.27 to -0.31, *p*-trend<0.000001) implies that ABSI may serve as a valuable metric for identifying individuals susceptible to accelerated BMD decline, particularly among metabolically obese yet normal-weight individuals. The observed inverted “J”-shaped relationship and threshold effect suggest that a subset of individuals may exhibit heightened risk with minimal reliance on BMI classification. These findings support the broader applicability of ABSI in predicting skeletal health outcomes. In clinical practice, integrating ABSI into osteoporosis screening protocols could refine risk stratification, especially for those not flagged as high-risk by conventional metrics. Future prospective studies are essential to validate the predictive capacity of ABSI for fracture incidence and to explore targeted therapeutic interventions aimed at visceral adiposity.

## Data Availability

The original contributions presented in the study are included in the article/**Supplementary Material**. Further inquiries can be directed to the corresponding authors.
