# Supplementary material for: Association between A body shape index and bone mineral density in middle-aged and elderly adults: a retrospective analysis of NHANES 2005–2018
Source: Front Endocrinol (Lausanne). 2025 Apr 7;16:1506841. doi: 10.3389/fendo.2025.1506841 (PMC12009725; doi:10.3389/fendo.2025.1506841)
Supplement: Supplementary file 1 [file Table1.docx]

Supplementary Material

## Ethical date and lot number


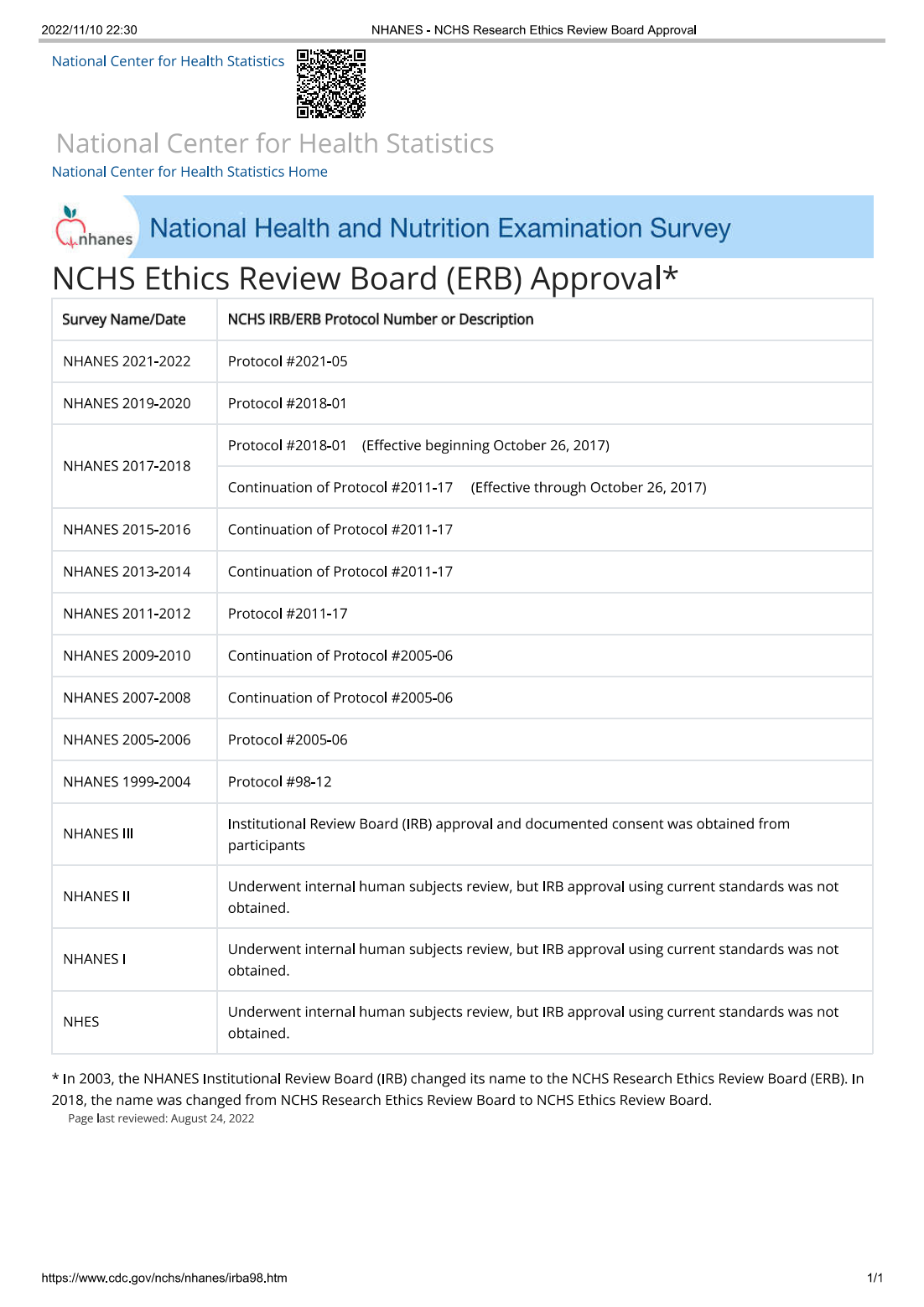


## 2. Missing rates of relevant covariates

| covariate | Smoking status | Diabetes | Hypertension | Alcohol consumption | Physical activity |
| --- | --- | --- | --- | --- | --- |
| missing value | 0 | 0 | 0 | 9519 | 1902 |
| missing rate | 0 | 0 | 0 | 76.64% | 15.31% |
